# Le polype sphénochoanal: une entité rare avec revue de la littérature

**DOI:** 10.11604/pamj.2020.37.139.20832

**Published:** 2020-10-08

**Authors:** Mounji Houda, Eddafi Salk, Benfdil Malika, Elfakiri Mehdi, Rochdi Youssef, Nouri Hassan, Raji Abdelaziz

**Affiliations:** 1 Service d’Otorhinolaryngologie et de Chirurgie Cervico-Faciale, CHU Mohammed VI de Marrakech, Marrakech, Maroc

**Keywords:** Polype sphénochoanal, chirurgie endoscopique, sinus sphénoïdal, Sphenochoanal polyp, endoscopic surgery, sphenoid sinus

## Abstract

Le polype sphénochoanal est une forme rare de polypes choanaux qui provient du sinus sphénoïdal et s'étend vers la choane via le récessus sphéno-ethmoïdal. Nous rapportons un cas de polype sphénochoanal chez un jeune adolescent de 15 ans qui s´est présenté pour un tableau d´obstruction nasale chronique évoluant depuis 3 ans, traité chirurgicalement par voie endoscopique avec une bonne évolution post-opératoire. L´incidence du polype sphénochoanal est extrêmement rare. Symptomatiquement, il ressemble à un polype antrochoanal. Ainsi, une évaluation préopératoire adéquate avec l´examen endoscopique et la tomodensitométrie ou l´imagerie par résonance magnétique est nécessaire pour un diagnostic précis et pour une stratégie chirurgicale appropriée. Le traitement de choix est l´exérèse chirurgicale par voie endoscopique.

## Abstract (en)

Sphenochoanal polyp is a rare form of choanal polyp originating from the sphenoid sinus and extending to the choanae via the sphenoethmoidal recess. We report a case of sphenochoanal polyp in a 15-year-old boy presenting with a 3-year history of chronic progressive nasal obstruction, treated with endoscopic surgery with good post-operative outcome. Sphenochoanal polyp is extremely rare. Symptomatically, it resembles an antrochoanal polyp. Adequate preoperative evaluation based on endoscopic examination and computed tomography (CT) scan or magnetic resonance imaging is necessary for accurate diagnosis and appropriate surgical strategy. Endoscopic excision is the treatment of choice.

## Introduction

Les polypes sphénochoanaux constituent une entité rare provenant du sinus sphénoïdal. Ils peuvent être différenciés des polypes antrochoanaux par l´examen endoscopique, la tomodensitométrie (TDM) et/ou l´imagerie par résonance magnétique (IRM) nasosinusienne [1]. Ils sont d´étiologie incertaine. La plupart des cas ont été rapportés chez les adultes jeunes [2]. En raison de leur localisation relativement profonde, ils sont diagnostiqués tardivement avec une symptomatologie variée et parfois non spécifique [3]. La rareté des lésions isolées du sinus sphénoïdal et ses rapports anatomiques importants nécessitent une investigation de la pathologie sous-jacente, en particulier de la malignité, de l'origine et de l'étendue de la masse avant l'excision chirurgicale [4].

## Patient et observation

**Figure 1:**
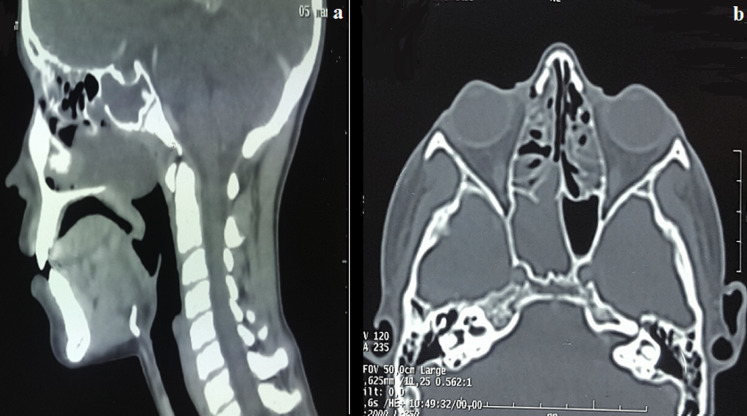
images scannographiques nasosinu siennes en coupe sagittale (a) et en coupe axiale (b) montrant un comblement hypodense homogène du sinus sphénoïdal étendu au cavum et à la fosse nasale droite

**Figure 2:**
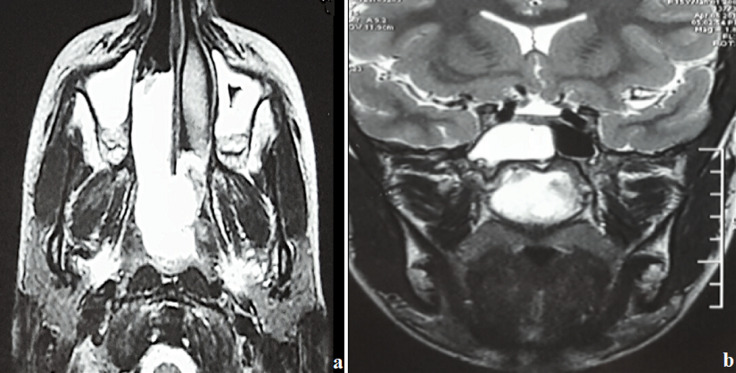
images d´IRM nasosinusiennes en coupe axiale (a) et en coupe coronale (b) montrant un comblement liquidien du sinus sphénoïdal droit étendu à la fosse nasale homolatérale

**Figure 3:**
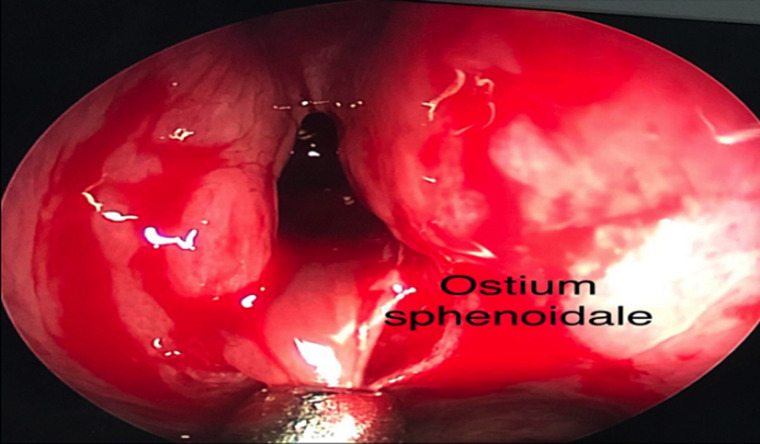
image endoscopique montrant la partie du polype émanant de l´ostium sphénoïdal

**Figure 4:**
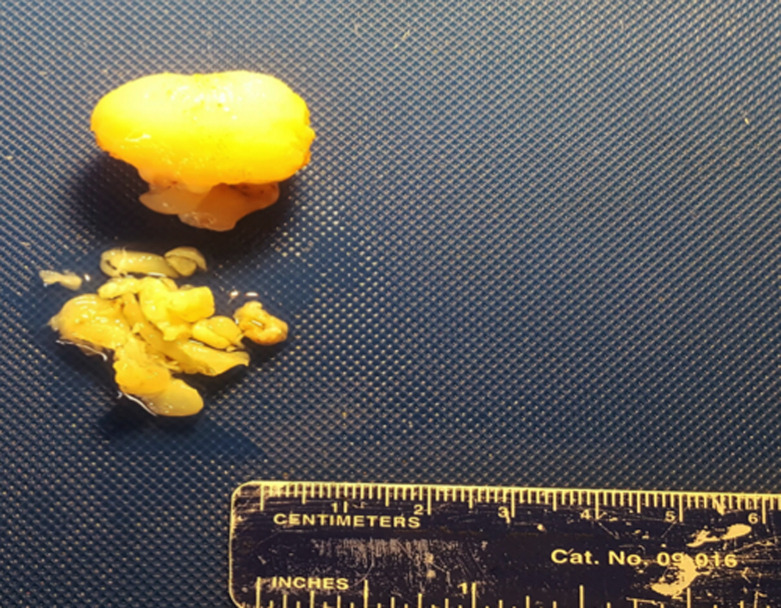
image montrant la pièce opératoire

Il s´agit d´un adolescent de 15 ans ayant comme antécédent une adénoïdectomie il y a 3 ans, qui se présente pour une obstruction nasale droite évoluant depuis 3 ans devenant permanente et bilatérale avec des rhinorrhées claires, anosmie et des céphalées faciales ethmoïdales sans épistaxis ni signes ophtalmologiques ou neurologiques. L´examen endoscopique a montré une formation polyploïde, kystique, lisse et régulière comblant la fosse nasale droite dont l´origine est difficile à déterminer. L´examen tomodensitométrique en coupes coronales et axiales a montré un comblement hypodense et homogène du sinus sphénoïdal élargissant le récessus sphéno-ethmoïdal étendu au cavum et la fosse nasale droite (Figure 1). L´imagerie nasosinusienne par résonance magnétique a montré un comblement liquidien du sinus maxillaire, des cellules ethmoïdales et du sinus sphénoïdal droits avec comblement de la fosse nasale droite (Figure 2). L´exploration chirurgicale a montré un polype translucide de la paroi postérieure de la fosse nasale droite émanant du récessus sphéno-ethmoïdal (Figure 3) dont la base d´implantation est au niveau de la paroi inférieure du sinus sphénoïdal. L´exérèse de la base d´implantation a été faite avec polypectomie et résection de la portion intrasinusienne (Figure 4). Les suites post-opératoires étaient simples. L´examen anatomopathologique définitif a montré un polype inflammatoire. Après 5 mois de suivi, le patient ne présentait plus aucun symptôme et l'endoscopie n'a révélé aucune récidive.

## Discussion

Le polype sphénochoanal est une entité clinique rare et bien moins fréquente que le polype antrochoanal. D'après les données de la littérature, il survient essentiellement chez les adolescents et les adultes jeunes (54,5% entre 10 et 20 ans selon Piquet *et al*. [5] bien que plusieurs observations rapportées dans la littérature retrouvent des patients plus âgés entre 50 et 70 ans [6, 7]. Il n'y a pas de prédominance de sexe. Histologiquement, les polypes choanaux sont similaires. Ils sont formés par un centre kystique entouré d'un stroma œdémateux fait d'infiltration de cellules inflammatoires. Sa surface est recouverte d'épithélium respiratoire où se trouvent des zones de métaplasie [8]. Chez notre patient, l´étude histopathologique de la pièce opératoire a révélé un polype inflammatoire. Le polype sphénochoanal doit être distingué de la méningo-encéphalocèle (présenté comme un défaut de la base du crâne permettant la communication entre la cavité cérébrale et nasale), le fibrome nasopharyngien (obstruction nasale et épistaxis unilatérale récidivante), le papillome inversé (une tumeur unilatérale avec l'élargissement du complexe ostéo-méatal chez les patients plus âgés) [1]. Cependant, son principal diagnostic différentiel reste le polype antrochoanal puisqu´ils partagent les mêmes aspects cliniques et histopathologiques [9].

La symptomatologie clinique des polypes sphénochoanaux est presque invariable de celle des polypes antrochoanaux, mais il existe des différences en termes de résultats de l'examen endoscopique et du scanner nasosinusien [9]. L´obstruction nasale constitue le maitre symptôme des patients atteints de polypes sphénochoanaux, elle peut être unilatérale ou bilatérale. Une rhinorrhée, des céphalées et parfois des ronflements peuvent également survenir [9, 10]. Dans notre cas, le principal symptôme était l´obstruction nasale unilatérale au début devenant bilatérale progressivement. En raison de leur localisation profonde, ces polypes risquent de s´échapper à la rhinoscopie antérieure, d´où l´intérêt de l'endoscopie nasale qui est très essentielle et obligatoire, non seulement pour confirmer le diagnostic, mais également pour visualiser l'étendue du polype. Cependant, la TDM des sinus est l´examen de référence [1, 7]. L'utilisation combinée de techniques d'imagerie et d'endoscopie nasale diagnostique est suggérée pour un diagnostic précis des lésions isolées du sinus sphénoïdal [9]. Le traitement des polypes sphénochoanaux est exclusivement chirurgical, essentiellement par la voie endoscopique endonasale [1, 10]. Cette voie offre une excellente vision des sinus impliqués et est associée à un faible taux de récidive par rapport à celui d'une simple polypectomie. Les autres avantages de cette technique incluent une déperdition sanguine minimale, douleur moindre, une récupération rapide et un temps opératoire plus court [5, 10].

## Conclusion

Le polype sphénochoanal est une cause d'obstruction nasale rare dont l'étiopathogénie précise reste inconnue. Cette rare affection nécessite un examen endoscopique, une tomodensitométrie et/ou une IRM des sinus pour une évaluation complète. Le traitement de référence reste la chirurgie endoscopique permettant une exérèse complète du polype diminuant ainsi le risque de récidive et l´incidence de complications possibles.
